# Significance of Specific Oxidoreductases in the Design of Hypoxia-Activated Prodrugs and Fluorescent Turn off–on Probes for Hypoxia Imaging

**DOI:** 10.3390/cancers14112686

**Published:** 2022-05-29

**Authors:** Ewelina Janczy-Cempa, Olga Mazuryk, Agnieszka Kania, Małgorzata Brindell

**Affiliations:** 1 Department of Inorganic Chemistry, Faculty of Chemistry, Jagiellonian University in Krakow, Gronostajowa 2, 30-387 Krakow, Poland; ewelina.janczy@doctoral.uj.edu.pl (E.J.-C.); olga.mazuryk@uj.edu.pl (O.M.); 2 Institute of Biology, Pedagogical University of Krakow, Podchorążych 2, 30-084 Krakow, Poland; agnieszka.kania@up.krakow.pl

**Keywords:** hypoxia, hypoxia-activated prodrugs, hypoxia-inducible factor 1, tumor microenvironment, fluorescent probes, hypoxia imaging, oxidoreductases, nitroreductase, cytochrome P450 reductase, quinone oxidoreductase, xanthine oxidase, azoreductase, cytochrome b_5_ reductase

## Abstract

**Simple Summary:**

Hypoxia-activated prodrugs (HAPs), selectively reduced by specific oxidoreductases under hypoxic conditions, form cytotoxic agents damaging the local cancer cells. On the basis of the reported clinical data concerning several HAPs, one can draw conclusions regarding their preclinical attractiveness and, regrettably, the low efficacy of Phase III clinical trials. Clinical failure may be explained, inter alia, by the lack of screening of patients on the basis of tumor hypoxia and low availability of specific oxidoreductases involved in HAP activation. There is surprisingly little information on the quantification of these enzymes in cells or tissues, compared to the advanced research associated with the use of HAPs. Our knowledge about the expression and activity of these enzymes in various cancer cell lines under hypoxic conditions is inadequate. Only in a few cases were researchers able to demonstrate the differences in the expression or activity of selected oxidoreductases, depending on the oxygen concentration. Additionally, it was cell line dependent. More systematic studies are required. The optical probes, based on turning on the fluorescence emission upon irreversible reduction catalyzed by the overexpressed oxidoreductases, can be helpful in this type of research. Ultimately, such sensors can estimate both the oxidoreductase activity and the degree of oxygenation in one step. To achieve this goal, their response must be correlated with the expression or activity of enzymes potentially involved in turning on their emissions, as determined by biochemical methods. In conclusion, the incorporation of biomarkers to identify hypoxia is a prerequisite for successful HAP therapies. However, it is equally important to assess the level of specific oxidoreductases required for their activation.

**Abstract:**

Hypoxia is one of the hallmarks of the tumor microenvironment and can be used in the design of targeted therapies. Cellular adaptation to hypoxic stress is regulated by hypoxia-inducible factor 1 (HIF-1). Hypoxia is responsible for the modification of cellular metabolism that can result in the development of more aggressive tumor phenotypes. Reduced oxygen concentration in hypoxic tumor cells leads to an increase in oxidoreductase activity that, in turn, leads to the activation of hypoxia-activated prodrugs (HAPs). The same conditions can convert a non-fluorescent compound into a fluorescent one (fluorescent turn off–on probes), and such probes can be designed to specifically image hypoxic cancer cells. This review focuses on the current knowledge about the expression and activity of oxidoreductases, which are relevant in the activation of HAPs and fluorescent imaging probes. The current clinical status of HAPs, their limitations, and ways to improve their efficacy are briefly discussed. The fluorescence probes triggered by reduction with specific oxidoreductase are briefly presented, with particular emphasis placed on those for which the correlation between the signal and enzyme expression determined with biochemical methods is achievable.

## 1. Introduction

Hypoxia generally refers to low oxygen tissue levels (<5–10 mmHg) and is an important characteristic of most solid tumors and hematological malignancies [1]. All solid tumors are subject to hypoxia contain aerobic cells, and some also contain hypoxic cells, however there is substantial variation in the fraction of hypoxic cells and its severity [2]. The hypoxic state is caused by the imbalance between the oxygen supply to the cells and its consumption rate. Several types of tumor hypoxia are identified, including acute or chronic hypoxia. This classification is based on empirical observations and could be an oversimplification of the real picture [3]. Chronic (or diffusion-limited) hypoxia usually refers to reduced oxygen concentration due to fast tumor expansion and inadequate blood-vessel formations. Chronic hypoxic changes contribute to high-frequency DNA breaks and the accumulation of DNA errors leading to mutagenesis. Acute (or perfusion-related) hypoxia is mainly caused by local, temporary disturbance in blood perfusion due to physical obstruction (e.g., cell aggregation) or transient vascular collapse. Acute hypoxia could lead to a generation of high levels of reactive oxygen species (ROS), decreased oxygen metabolism, and an activation of autophagy.

The response of cells to hypoxic conditions depends in part on the duration and severity of exposure to hypoxia [4]. Primarily, hypoxia leads to the impairment of the proliferative capacity of cells and eventually to cellular death. However, a minority of tumor cells may adjust to hypoxic stress and survive by triggering proteomic and genomic modifications, leading to the growth stasis or impairment through cell-cycle arrest, differentiation, programmed cell death, or necrosis [3]. One of the most important factors that mediate these processes is the oxygen-sensitive transcription factor HIF-1 (hypoxia-inducible factor 1), which activates a battery of more than 30 genes involved in various cellular pathways. The mechanism of its activation is described in the next paragraph. Hypoxia is found to be a driving force for tumor angiogenesis. Additionally, oxygen deficiency is associated with a substantial downregulation of cell adhesion molecules (E-cadherin and integrins), which in turn causes cell detachment and the induction of spontaneous metastasis [5]. Hypoxia can lead to the development of a more aggressive tumor phenotype and can explain the delayed recurrences, dormant micrometastases, and growth retardation observed in large tumors [6]. Several clinical studies have identified hypoxia as a negative prognostic indicator for patient outcomes [2,7]. It should be noted that hypoxia is not only a feature of macroscopic tumors. Studies show that vascular micrometastases (<1 mm in diameter) were severely hypoxic [8].

In addition to poor tumor prognosis, hypoxia is associated with the resistance to various nonsurgical anticancer therapies [3,9]. Due to the diminished oxygen availability, hypoxia directly reduces the efficiency of oxygen-related treatments, such as standard radiotherapy, O_2_-dependent chemotherapy (e.g., bleomycin and doxorubicin), and photodynamic therapy. The activity of a broad range of cytotoxic drugs (carboplatin and cyclophosphamide) was found to be oxygen-dependent, due to the increased activity of DNA-repair enzymes and decreased cellular proliferation caused by hypoxic conditions. Studies also reported a diminished response of hypoxic tumors to chemo-, immune-, and hormonotherapy due to aberrant tumor microvascular and induced post-transcriptional modifications. Although several clinical studies have been published that demonstrate a significant direct correlation between tumor hypoxia and poor clinical outcomes after radiotherapy [10,11,12], the hypothesis that hypoxia limits the curability of human cancer by other methods is based on numerous different in vitro studies [5]. However, there is emerging evidence that hypoxia markers can be used to guide cancer patient therapy. Several independent trials showed that patients with hypoxic tumors experienced greater benefits from hypoxia-modifying radio- and chemotherapy, as well as anti-angiogenic therapy [13]. The heterogeneity of hypoxia in tumors due to the fluctuation of hypoxia with time (cycles of hypoxia–normoxia) or steep spatial oxygen gradients (4D heterogeneity) appears to be extremely relevant for clinical outcomes [3]. Furthermore, the existence of macroscopic regional hypoxia is characterized by the presence of large numbers of apparently functional vessels; however, individual vessels and their branches remain unpaired that result in extended longitudinal gradients of nutrients, especially including oxygen [14].

This picture is additionally complicated by the different adaptive and genetic consequences of hypoxia between and within tumor types [13].

Being a severe negative factor, hypoxia represents a unique tumor vulnerability, which provides an opportunity for tumor-selective therapies [4,15]. Increased efforts have been made to develop therapeutic agents that selectively target hypoxic cells. Hypoxia-activated prodrugs (HAPs) are compounds that are activated by selective reduction with specific oxidoreductases under hypoxic conditions to form cytotoxic compounds. In this way, the drugs selectively target hypoxic cancer cells and show no toxicity to healthy tissues. Additionally, HAPs being active towards hypoxic micrometastasis have the potential to prevent such metastasis from developing into macroscopic tumors, thus decreasing the metastatic rate of the tumors [1]. Since therapeutic efficiency is notoriously compromised by the hypoxic conditions of the tumor microenvironment, the accurate monitoring of oxygen levels can bring additional information to clinicians and provide significant therapeutic benefits [16]. In preclinical and clinical studies, in addition to direct oxygen measurements using needle-type O_2_ electrodes, many indirect methods requiring surgical resection or biopsy applying immunolabeling have been developed. Furthermore, techniques that offer direct read-outs, such as Magnetic Resonance Imaging (MRI) or Positron Emission Tomography (PET) and Optical Imaging (OI), particularly those based on fluorescent probes, have been extensively investigated. Many excellent reviews present the recent findings in this field [14,17,18,19]. In many cases, probes for PET and OI relay on selective activation under hypoxic conditions of the tumor microenvironment ensuring not only reduced oxygen concentration, but also increased expression or activity of the relevant oxidoreductases [14,20,21].

In this review, we refer to the current knowledge of oxidoreductases, which are prerequisites for successful hypoxia-activated prodrug therapies, as well as for fluorescent turn off–on probes designed to assess hypoxia that are non-florescent (off) and restore fluorescent properties (on) due to reduction by appropriate oxidoreductases. We also briefly discuss the current clinical status of HAPs and the possibilities to improve their efficacy. Additionally, the designed strategy for fluorescent probes is briefly presented along with examples of such probes, for which their signals are correlated to oxidoreductase expressions determined with biological methods.

## 2. Hypoxia-Inducible Factor 1—Adaptive Response to Hypoxia

**Figure 1:**
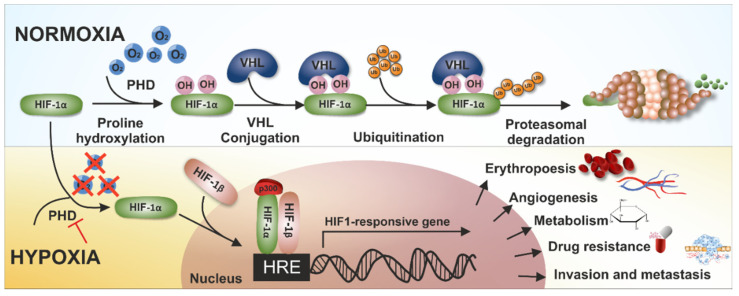
Mechanism of HIF-1 regulation in tumor cells.

Hypoxia-inducible factor 1 (HIF-1) is a transcription factor that is expressed by all cellular organisms and has become known as the main regulator of oxygen homeostasis [22,23]. HIF-1 is a heterodimeric protein composed of an inducible α-subunit (HIF-1α) and constitutively expressed β-subunit (HIF-1β). HIF-1α is an oxygen-sensitive subunit and its expression is precisely controlled in cells by post-translational modifications. The mechanism regulating the level of the active form of the HIF-1α subunit is based on oxygen-dependent hydroxylation. HIF-1α hydroxylation involves two enzymes, prolyl hydroxylase (PHD) and asparaginyl hydroxylase (FIH), which, in the presence of oxygen as well as the ascorbate and iron ions as cofactors, introduce a hydroxyl group to a proline or asparagine residue, at the same time oxidizing α-ketoglutarate into succinate [24]. PHD hydrolyzes two proline residues, which are located in the oxygen-dependent degradation (ODD) domain in HIF-1α, leading to the binding of the hydroxylated domain to von Hippel–Lindau (VHL) proteins. The binding of the HIF-1α subunit to the VHL protein activates the ubiquitination and targeting of HIF-1α to proteasomal degradation [25]. Under hypoxic conditions, proline hydroxylation cannot be activated due to the lack of oxygen as a substrate; hence, the accumulation of HIF-1α and the consequent activation of HIF-1 occurs. Moreover, there is a second mechanism of the negative regulation of the HIF-1α pathway under aerobic conditions. FIH in the presence of oxygen hydrolyzes an asparagine residue in the C-TAD (C-terminal transcriptional activation domain) in the α-subunit of HIF-1. As a consequence, the interaction with one of the essential cofactors, p300/CBP, is inhibited, preventing HIF activation [26]. In hypoxia, HIF-1α is protected from degradation and accumulates in the cytoplasm, and then enters the nucleus where it dimerizes with the constitutively expressed HIF-1β. The heterodimer binds to hypoxia-response elements (HREs) that initiate the transcription of target genes [27,28] (Figure 1).

The activation of complex intracellular signaling pathways through HIF-1 enables cellular adaptation to hypoxic stress. In order to adapt metabolism to a low oxygen concentration, HIF-1 induces the expression of genes associated with the activation of glycolytic metabolism that results in the shift from oxidative phosphorylation to anaerobic glycolysis and stimulates glucose conversion into glycogen, ensuring energy storage to survive prolonged stress. Since the glycolytic pathway is less efficient, hypoxic cells tend to consume more glucose to meet their energy needs. Furthermore, HIF-1 is involved in the transcriptional activation of pro-angiogenic factors as well as other vascular endothelial growth factors (VEGFs) engaged in the development of new blood vessels. At the same time, the overexpression of HIF-1 has a profound effect on each stage of the metastasis cascade and promotes the formation of a more aggressive tumor phenotypes by the transcriptional activation of oncogenic growth factors [7,28,29]. To date, the expression of many gene-encoding proteins relevant to cancer biology was found to be regulated by HIF-1, as recently reviewed [28,30,31,32].

Due to the multifactorial effect of HIF-1, its expression is well correlated with tumor malignancy and the level of tissue hypoxia; therefore, HIF-1 is an exceptional therapeutic marker. However, due to the lack of standardization of the immunohistochemistry protocol and interpretation of the results, this exogenous marker is difficult to apply, and the studies conducted confirmed the correlation between this parameter and the measured oxygen concentration [33].

Apart from the key role of the HIF-1α subunit in regulating hypoxia response, HIF-2α has an equally important and very specific function. Despite their regulatory, structural, and functional similarities, the two entities have significantly different tissue-specific expression patterns, and their effect on the expression of some genes is different [34]. HIF-1α is present in all tissues in the organism, while HIF-2α expression is restricted to specific tissues. HIF-2α promotes the hypoxic induction of erythropoietin (EPO), and induces genes responsible for metastasis, especially matrix metalloproteinases (MMPs) and a stem cell factor. HIF-2α is stabilized and activated at higher oxygen tension levels than HIF-1α, which makes HIF-1α the main driver of the acute hypoxia response, and HIF-2α is responsible for adaptive mechanisms in chronic hypoxia [35].

## 3. Hypoxia-Activated Prodrugs (HAPs)

Hypoxia-activated prodrugs (HAPs) are regarded as bioreductive drugs that are selectively activated under hypoxic conditions and that can precisely target the hypoxic regions of solid tumors, which are an important cause of treatment resistance to conventional therapies. Under such conditions, these compounds can be selectively reduced by specific oxidoreductases to form cytotoxic agents that accurately target hypoxic tumor cells, while having only little toxicity to normal tissue. Among the representatives of these drugs, there are quinones, nitroaromatics, aliphatic N-oxides, and hetero-aromatic N-oxides [1]. In the case of most of HAPs (e.g., nitroaromatics, quinones, and benzotriazine di-oxides), the process of their activation in tumors is initiated by an irreversible enzymatic one-electron reduction by flavin-dependent oxidoreductases, leading to the generation of a prodrug radical anion. In well-oxygen-saturated tissues, it is quickly scavenged by molecular oxygen. However, under hypoxic conditions, the radical anion either becomes fragmented or further reduced, forming cytotoxic effector species that interact with a pharmacological target. As a result, the alkylation and damage of DNA, the inhibition of kinase, or inactivation of topoisomerase II occur, which leads to the hypoxic cell’s death. There is also a group of HAPs (e.g., some quinone and nitroaromatic compounds, and aliphatic N-oxides, such as banoxantrone) that are activated via two-electron reduction [36].

**Figure 2:**
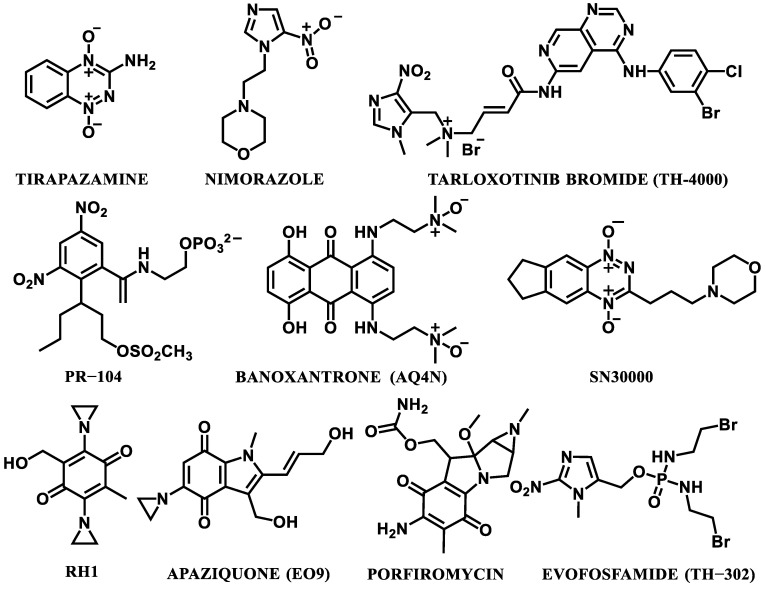
Structures of the selected hypoxia-activated prodrugs (HAPs) and hypoxic radiosensitizer: nimorazole.

The design process of and investigation into resultful HAPs have been conducted for about 50 years and, for this study, over a dozen potential compounds have been approved for the clinical trials. According to our knowledge, there have been about ten of these compounds under extensive preclinical and clinical evaluations, e.g., tirapazamine, apaziquone/EO9, banoxantrone, porfiromycin, PR-104, RH1, evofosfamide/TH-302, SN30000, and tarloxotinib bromide/TH-4000, as well as nimorazole, not exactly HAPs, but used as a hypoxic radiosensitizer in radiotherapy (Figure 2). Only five of them (TH-302, porfiromycin, EO9, tirapazamine, and nimorazole) reached Phase III; however, none of them have achieved regulatory approval yet [1,36,37,38]. The summary of the clinical studies is presented in Table 1.

TPZ (tirapazamine, 3-amino-1,2,4-benzotriazine-1,4dioxide) was the first evaluated HAP. It was reported in 1986 [51], and its clinical safety was confirmed in 1994 [37,52]. The main catalytic reductase involved in the reduction of TPZ is cytochrome P-450 [1,53]. The mechanism of action of this prodrug concerns the damage of both purine and pyrimidine residues in double-stranded DNA, which leads to its break, chromosome aberrations, and hypoxic cell death [1]. However, at the stages of Phase I and II clinical trials, TPZ seemed to be a very promising agent, having satisfactory antineoplastic efficacy and tolerable toxicity; the subsequent Phase III clinical studies revealed more significant disadvantages (e.g., muscle cramping, ototoxicity, granulocytopenia, nausea, and vomiting) [1]. As it was reported, a number of analogues of TPZ have been developed, however none of them have been investigated at the clinical stage [37,54].

TH-302 (evofosfamide) is a second-generation HAP. It consists of a 2-nitroimidazole moiety linked to bromo-iso-phosphoramide mustard (Br-IPM), which alkylates DNA. The cytotoxic effect occurs under hypoxic conditions through a 2-nitroimidazole reduction reaction and the release of Br-IPM. The reduction reaction is determined by cytochrome P450 oxidoreductase. The efficacy of TH-302 is highly dependent on the tumor type [1,55]. This HAP has been tested both as monotherapy as well as in different combinations with existing anticancer therapies (e.g., chemo- or radiotherapy). Peeters et al. reported a causal relationship observed between the tumor oxygenation levels and the therapeutic efficacy [56]. In case of combination therapies, the treatment should be precisely planned, since the increased hypoxia may oppose the effects of chemo- or radiotherapy [37]. Despite the very promising results, clinical safety, and therapeutic efficacy confirmed in Phase I and II clinical studies [57,58,59,60], TH-302 did not achieve the positive results in Phase III, similar to TPZ. However, further investigation is in progress [1].

TH-4000 (tarloxotinib) is nowadays one of the most clinically advanced molecularly targeted HAPs, a bioreductive pan-HER inhibitor. The mechanism of action of this prodrug under hypoxic conditions involves its 1-electron reduction to a nitro radical anion that afterwards fragments and releases an irreversible epidermal growth factor receptor (EGFR) tyrosine kinase inhibitor (TKI) [38,61]. Unfortunately, the clinical trials in Phase II with TH-4000, as reported by Mistry et al. [38], were terminated because of the unsatisfactory results [32].

One of the promising compounds is nimorazole. It belongs to a class of chemicals known as 5-nitroimidazoles. What is crucial is that this drug makes tumor cells more sensitive to radiotherapy. Currently, it is in the Phase III of the clinical trials (NCT01880359). The efficiency and safety of this substance has already been proved and reported [44,62,63]. As it was mentioned by Tharmalingham, H. et al. [64], the use of nimorazole as a radiosensitizer was a standard practice in Denmark alone, as Danish research confirmed its safety in the treatment of, e.g., supraglottic and pharynx tumors. Recently, new clinical trials with nimorazole in Denmark and the UK have also been reported, but it is still necessary to assess whether this drug directly changes the oxygenation status of patient tumors [65].

The interest in this promising group of compounds has also led to the recommencement of research into a new class of oxygen-mimetic nitroimidazole sulfonamide radiosensitizers [66]. This confirms that the development of HAPs is essential and gives hope for medical success.

Despite the confirmed preclinical attractiveness and antineoplastic effects of several investigated HAPs, their limitations and deficiencies at the clinical stage have also been revealed by many studies [1,35,36,38]. What is crucial is that none of the clinical trials in Phase III did not take into account the assessment of the levels of tumoral hypoxia, and this factor may be critical since these levels greatly differ [37,67,68]. Therefore, there is a strong need for proper stratifications of patients according to the tumor hypoxia level at the clinical-trials stage (e.g., using a combination of biomarkers to identify patients with hypoxic tumors and biomarkers for specific prodrug-activating oxidoreductases) [37].

Furthermore, Mistry et al. indicated the lack of optimization of HAP delivery to the remote target cell from the functional vessels as the next reason of failure of these prodrugs. Another problem also pointed out by them was the limitation resulting from the overlapping toxicity of both HAPs and a chemotherapeutic agent used in combined therapy forcing a reduction in therapeutic doses of drugs [38].

Generally, the following directions for overcoming these drawbacks, or at least their improvement, may be undertaken:

1. The development of predictive biomarkers for identifying the oxidoreductase enzymes involved in the reaction of catalysis of the activation of HAPs via electron donation, as well as the development of response biomarkers [36,37,38,69].
2. The development of screening methods (e.g., PET/CT imaging) for the selection of the best potential prodrugs [1].
3. The advancement of combined methods, e.g., with chemotherapy, radiation therapy, photodynamic therapy (PDT), or starvation therapy [1,38,70].
4. The development of HAPs that are preferably activated to release molecularly targeted protein ligands, rather than DNA-damaging cytotoxins, in order to limit the toxicity effects [38].
5. Implementation of a personalized therapeutic approach to rationally select an optimal HAP for an individual tumor, taking into account the tumor hypoxia level [36,37].
6. Further development of gene-directed enzyme-prodrug therapy (GDEPT). This kind of therapy engages an enzyme–prodrug combination in order to generate high levels of bystander cell killing [71]. A genetically encoded therapeutic enzyme is indirectly delivered to the tumor milieu, and this process is mediated by a tumor-tropic bacterial or viral vector. Subsequently, the enzyme transforms the delivered non-toxic prodrug into a potent cytotoxin and the therapeutic effect is much stronger, compared to non-targeted traditional agents [72]. Enzymes, particularly bacterial nitroreductases, known to activate anticancer nitroaromatic prodrugs, are very promising for their use in GDEPT [73,74].

The convergence of these two factors, namely, tumor hypoxia and oxidoreductase expression, seems to be crucial for the further development and understanding of the current failures of HAP therapies. Thus, we focused on these aspects and their relationship to each other.

## 4. Oxidoreductases

One of the main mechanisms of cell adaptation to hypoxia is the switch from oxidative phosphorylation to glycolysis, which results in an increased production of NADH. NADH is the main source of electrons in the cell. Therefore, the imbalance between NAD+ and NADH changes the cellular redox potential and creates a more reducing environment, compared to normal tissues [75]. These microenvironmental conditions favor the overexpression of many redox enzymes, such as nitroreductases (NTRs), azoreductases, cytochrome p450 reductase, and xanthine oxidase [76,77,78,79]. As previously mentioned, one of the approaches in designing oxygen-sensitive prodrugs that selectively target hypoxic tumor cells is taking advantage of the overexpression of these oxidoreductases in hypoxia [1]. Additionally, in recent years, this metabolic feature was exploited to design small molecule probes for hypoxia imaging. Unfortunately, the identification of HAP-activating enzymes, their expression in neoplastic tissues, and their potential as sensitive biomarkers of hypoxia have not yet been resolved. There is surprisingly little information on the quantification of these enzymes in cells or tissues, compared to the advanced research associated with the use of HAPs. Studies comparing the expression of these enzymes under normoxic and hypoxic conditions are rare. The table below (Table 2) summarizes the available information about the quantitative or semi-quantitative determination of selected oxidoreductases. In the following chapters, individual enzymes are discussed, with particular emphasis being placed on their quantitative and semi-quantitative assessments in vitro (or in vivo), under various oxygen conditions.

### 4.1. Nitroreductases (NTRs)

**Figure 3:**
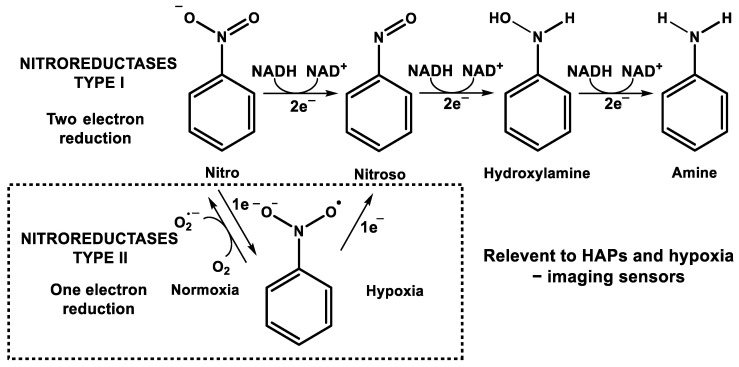
The mechanism of reduction of nitroaromatic compounds by type-I and -II nitroreductases.

Nitroreductases (NTRs) are a group of enzymes that catalyze the reduction of nitroaromatic compounds using NAD(P)H as the reducing agent and flavin mononucleotide (FMN) or flavin adenine dinucleotide (FAD) as the prosthetic group [91]. In recent years, NTRs have gained great interest due to their use in bioremediation, antibiotics therapy, and activation of prodrugs in targeted anticancer therapies [92]. NTRs have been grouped into two categories, depending on the occurrence of one- or two-electron reduction and sensitivity to oxygen [93]. Type-I NTRs, which are oxygen-insensitive, catalyze the two-electron transfer from NAD(P)H to the nitro group proceeding through nitroso and hydroxylamine intermediates to the fully reduced amine form. The reduction process catalyzed by NTR type I is independent of the oxygen concentration. The reduction of nitroaromatic compounds is based on the ping-pong mechanism. In the first stage, the oxidation of NAD(P)H occurs with the transfer of hydride to the flavin system, and then the substrate is reduced with the simultaneous re-oxidation of the flavin. Type I of the NTRs occurs in bacteria and fungi, with the nitroreductase from *Escherichia coli* being the best studied [94]. NAD(P)H quinone dehydrogenase 1 (NQO1) can be considered as type-I NTR functioning in the mammalian system. In contrast, type-II NTRs are oxygen-sensitive and function only in extreme hypoxia environments. Type-II NTRs catalyze the one-electron reduction process, generating the nitro anion radical, which, in the presence of oxygen, is rapidly re-oxidized to the original nitro-aromatic compound, simultaneously producing the peroxide radical. This creates a “futile redox cycle”, which can cause oxidative stress [21,91]. However, in an anaerobic environment, re-oxidation does not occur and the reduction of the nitro group is complete and irreversible [79] (Figure 3). Type-II NTRs are mainly found in mammalian systems and, due to their selective action in hypoxia, are of particular significance to the design of prodrugs and hypoxia-sensitive imaging sensors. Different type-II nitroreductases are associated with the cytoplasm, mitochondria, and microsomes, and they include cytochrome p450 reductase (POR), xanthine oxidase (XO), or cytochrome b_5_ reductase (CYB5R). In many studies, there is no clear indication of which nitroreductase is tested, and these works are discussed in this chapter, while the following chapters present data from the studies with individual enzymes.

Nitroreductases are a widely studied therapeutic target for the activation of prodrugs targeting cancer cells. Moreover, it was suggested that the assessment of the NTR level could be directly employed to assess the level of hypoxia, which would help to define the disease state and the treatment prognosis [21]. Despite proposing the level of NTR in cells as an effective biomarker of hypoxia, the data on the direct relationship between the concentration of NTR and the degree of hypoxia are contradictory [79,80]. Sh. Luo et al. showed that in three cancer cell lines, namely, HepG-2, A549 and SKOV-3, the NTR concentration in the same cell line quantified using the ELISA test remained at a similar level at different O_2_ concentrations ranging from 20–0.1%, reached by growing cells for 8 h in AnaeroPack (Mitsubishi Gas Corp., New York, NY, USA) (Table 1) [80]. Furthermore, the quantification of the expression of NTR in solid HepG-2 tumors (in a mouse model) indicated that the NTR levels remained unchanged with tumor growth and age [80].

Recently, our group focused on a new group of nitro-pyrazinotriazapentalene derivative sensors as fluorescent turn off–on probes for imaging NTRs levels in cancer cells [79]. The quantification of NTRs was performed in the human highly invasive melanoma cell line A2058. We demonstrated that, after 24 h of incubation, cells in a hypoxia chamber filled with a gas mixture comprising 94% N_2_, 5% CO_2_, and 1% O_2_, the level of NTR in cells increased from 180 to 300 pg/mL (determined in 1 mg of protein per ml of cell lysates), as detected by the ELISA test (see Table 1). Moreover, the chemical induction of hypoxia using DFO (200 µM for 24 h) increased the level of protein in cell lysates. The level of protein expression was strongly correlated with the activation of the investigated nitroaromatic probes, demonstrated by the increase in the level of fluorescence in the cells.

In 2022, a paper on the NTR-activated near-infrared probe was presented by Zhang et al. [81]. The research was conducted on the HeLa cell line, for which the NTR concentration, as detected by the ELISA test, significantly depended on the level of hypoxia. The eight-hour cell incubation under various atmospheres of oxygen resulted in an increase in NTR expression proportional to the oxygen concentration. The 15, 10, and 1% O_2_ concentrations corresponded to 2-, 5-, and 10-fold increase in NTR concentrations in cells, compared to one kept under 20% O_2_.

The above results were also confirmed by the in vivo studies conducted on the A549 tumor in the murine model by Y. Li et al. [82]. In this study, the presence of hypoxia in the A549 tumor in the mouse was confirmed using PET imaging. This result was further correlated with the level of nitroreductases assessed by Western blot detection. The band corresponding to the enzyme’s molecular weight was detected only in the neoplastic tissue. Furthermore, the enhancement of the fluorescence of the hypoxia-sensitive near-infrared (NIR) dye corresponded to the level of hypoxia in tumors of various sizes.

Another possibility of determining nitroreductase in cells involved carbonic anhydrase 9 (CA9) as a surrogate marker [83]. It was already shown that the expression of CA9 was induced by hypoxia, and it was generally considered to be possibly correlated with the oxygenation level [95]. However, as it has been recently determined [96] that it is dependent on a cancer cell line and a cancer type. K. S. Hettie et al. reported a positive correlation between oxygen-deprivation levels and total CA9 expression in a panel of four glioblastoma cell lines [83]. Western blot analysis showed that U87, U251, GBM2, and GBM39 cells grown 24 h in 2% O_2_ showed a 2-, 4-, 4-, and 8-fold increase in CA9 expression, compared to physiological conditions (20% O_2_), respectively. Furthermore, the applied fluorescent probe activated by NTR exhibited a similar increase in emission intensity (8-fold) upon incubation with GBM39 cells under the same conditions (2 vs. 20% O_2_). Based on this observation, a correlation between the expression of CA9 and NTR was suggested. More research is needed to observe if the demonstrated correlation is line-specific or can be applied more universally.

### 4.2. Azoreductases

Azoreductases are a diverse group of flavin-containing enzymes widely present in bacterial and higher eukaryotic organisms, which perform the reactions of reductive cleavage azocompounds [97]. These enzymes, such as NTRs, require NAD(P)H as an electron donor for reduction. Azoreductases are mainly responsible for the biotransformation and detoxification of azo and nitroaromatic dyes in the industry. However, in recent years, the interest in azoreductases has increased due to their use in drug activation. Pro-azodrugs are primarily used to selectively deliver drugs to the intestines, where they are cleaved by azoreductases secreted by the intestinal microflora. Thus, anti-inflammatory drugs, antibiotics, and anticancer drugs are delivered [98,99]. It has been shown that these enzymes, apart from the reduction of azocompounds, also reduce many other substrates, including quinones and nitroaromatic compounds. The reduction of nitrofuran antibiotics by azoreductases is widely studied [100], which presents great opportunities to design new prodrugs using this enzyme for activation.

The group of azoreductases also includes mammalian enzymes, such as NAD(P)H quinone dehydrogenase 1 (NQO1) and N-ribosyldihydronicotinamide:quinone reductase 2 (NQO2), which can reduce the same compounds as bacterial azoreductases [101]. NQOs are dimeric proteins that contain FAD as a cofactor in each subunit and catalyze the beneficial two-electron reduction of quinones to hydroquinones [102,103]. The DT-diaphorase catalyzed reduction prevents the undesirable reversible one-electron reduction of quinones to semiquinone with the generation of ROS. NQO1, which is better characterized than NQO2, has several important biological functions, including the detoxification of quinone compounds, protein stabilization, and activation of endogenous oxidants, such as vitamin K, co-enzyme Q, and α-tocopherol [104]. NQO1 is involved in the degradation of many proteins and is often overexpressed in many types of cancer, which is associated with a poor prognosis. NQO1 level also correlates with hypoxia [105]. NQO1 stabilizes HIF1α by binding and preventing its interaction with PHD [106]. Many studies have reported that the increased reduction of azogroups occurs under hypoxic conditions, which may be an effective strategy for designing prodrugs and hypoxia probes [107,108,109,110,111,112,113].

Punganuru et al. examined NQO1 expression levels by Western blot in four cell lines; two tumor cell lines, A549 and H460; and two normal lines, IMR90 and HUVEC [84]. In tumor cells, the expression level of NQO1 was very high, while the normal lines did not express the protein. The level of NQO1 expression correlated well with the intensity of the fluorescent NIR probe based on the conjugation of the dicyanoisophorone fluorophore with the NQO1 substrate—quinonopropionic acid. Moreover, in vivo studies in a mouse model showed that NQO1 was present only in the tumor tissue and not in the other ones. Additionally, to validate the NQO1-specific activation of the tested probe, NQO1-positive (A549) and NQO1-negative (MDA-MB-231) tumors were generated in the same mouse. Following the intravenous administration of the tested probe, intense fluorescence was observed only for the NQO1-positive A549 tumor, whereas there was no discernible signal in the NQO1-negative MDA-MB-231 tumor.

Studies comparing NQO1 expression under hypoxic and normoxic conditions in different tumor lines provide contradictory information. The studies performed by O’Dwyer et al. showed a 4-fold increase in NQO1 mRNA in human HT29 cells incubated for 24 h under anaerobic conditions. It is worth noting that, however, hypoxia was achieved by exposing the cells to insufflation through needles with N_2_ [86]. Manley et al. examined 15 different tumor lines for NQO1 levels in normoxia and hypoxia (0.3% for 24 h) [85]. Western blot analysis showed that NQO1 levels ranged from high (H460, DU145, A549, and FaDu cells), through intermediate (9 L, Colo-205, HT-29, U251, and BxPC-3), to low (KM12, H522, and PC3 cells), while, in T47D and MDA-MB-231 cells, NQO1 was not detected. However, no differences were found in the level of NQO1 between cells grown under hypoxia and normoxia.

### 4.3. Cytochrome p450 Reductase (POR)

Various investigations show that cytochrome p450 reductase plays a crucial role in the catalysis of the reduction of bioreductive agents [114]. Cytochrome p450 reductase (POR) is a membrane-bound enzyme localized in the endoplasmic reticulum that transfers electrons from NADPH to cytochrome p450 or other heme proteins. POR is involved in the metabolism of drugs and steroid hormones and xenobiotics [115]. Cytochrome p450 reductase is a one-electron reductase that, apart from the reduction of the cytochrome P450 under physiological conditions, is the major factor in the activation of quinone and nitroaromatic compounds [116,117,118,119]. Cytochrome p450 reductases are attractive therapeutic targets to activate prodrugs in hypoxia. A strong correlation was observed between the expression of cytochrome p450 reductase and the bioactivation of tirapazamine [53,120] and PR-104A (the metabolite of PR-104 after the hydrolysis of the phospho–ester bond) [116] in hypoxia. Accordingly, POR plays an important role in activating prodrugs, and these indications come from experiments in which the overexpression of this enzyme is induced. Guise et al. additionally demonstrated that the inhibition of POR expression by 91% reduced the toxicity of PR-104A by 47%, which confirms that POR is one of the main, but not the only, reductase responsible for the hypoxic activation of this prodrug [116]. Furthermore, the antiproliferative potency of SN30000 under oxygen-deprived conditions was strongly increased by the overexpression of POR [121]. Likewise, genetic screening for enzymes required for HAP activity in hypoxia identified cytochrome p450 reductase as a major determinant of sensitivity to HAPs [122]. Although much work has been conducted to confirm the involvement of POR in HAP activation, there is still insufficient research to quantify it in unmodified tumor cells under normoxic and hypoxic conditions. Nytko et al. showed that the expression level of POR in head and neck squamous cell carcinomas (UT-SCC-14) and in a lung cancer cell line (A549) was not influenced by hypoxia, remaining on the same level as in normoxia (Table 1) [55]. However, it was dependent on a cell line. The level of POR expression in the UT-SCC-14 cell line was strongly decreased compared to the A549 cell line, which correlated well with the sensitivity of cells to evophosphamide. Furthermore, the downregulation of POR levels in transfected A549 cells reduced their sensitivity to evophosphamide. This demonstrates the importance of POR evaluation for the effective application of prodrugs activated by this enzyme. Much recent work has focused on investigating the use of POR to bioactivate various hypoxia imaging probes [123,124,125,126].

### 4.4. Xanthine Oxidase (XO)/Xanthine Oxidoreductase (XOR)

Xanthine oxidase (XO) can be formed from xanthine dehydrogenase (XDH), either by irreversible proteolysis or reversibly by thiol oxidation or phosphorylation as a post-translational regulation of XDH activity [127]. In many studies, scientists do not indicate what form of enzyme they work with or cannot distinguish between them, and use the name xanthine oxidoreductase (XOR), which includes both XDH and XO. XOR is widely distributed in a variety of species, including humans, and is mainly responsible for purine catabolism. XOR is a homodimeric metalloflavoprotein containing a molybdenum ion, one flavin adenine dinucleotide (FAD) cofactor, and two iron–sulfur redox centers in each subunit [128,129]. XOR catalyzes the oxidation of hypoxanthine to xanthine and then to uric acid. The key difference between these two forms of enzymes is the decreased affinity for NAD^+^ and the increased affinity for O_2_ of XO compared to XDH. Both XO and XDH can univalently reduce molecular oxygen to a superoxide radical or divalently form hydrogen peroxide; however, XO is considered as a major ROS producer [130,131]. The ROS formed under physiological conditions activate various signaling pathways, but when homeostasis is disturbed, it has a destructive effect on cells [132]. In recent years, many scientists have indicated that XO can perform the functions of nitroreductase by reducing nitro compounds with the simultaneous oxidation of xanthine or NADH [133,134,135]. Additionally, in vivo studies demonstrated that the level of XOR expression was related to the oxygen concentration in the tissues. Hypoxia upregulates XDH gene expression, as well as its conversion to XO in a cellular system [88,136].

Linder et al. confirmed that the total XOR activity (XO + XDH) increased under hypoxia conditions. The incubation of BEAS-2B cells under hypoxic conditions (3% O_2_) for 24 or 48 h increased XOR activity by 3- and 8-fold, compared to normoxic conditions (21% O_2_), respectively [87]. A further decrease in O_2_ concentration to 0.5% did not significantly change XOR activity, compared with 3%. However, the quantitative XOR protein analysis performed using the ELISA test, as well as Western blot, and the analysis of the XHD mRNA level in lysates showed no change regardless of the oxygen concentration. The increase in XOR activity in hypoxia without the need to synthesize new proteins was related to the post-translational activation of XOR by phosphorylation at low oxygen concentrations. This process was not associated with the conversion of XDH to XO. Two independent groups of researchers attained similar conclusions [89,90]. Kayyali et al. showed that XOR was phosphorylated in hypoxic RPMEC cells through a mechanism engaging p38 kinase and casein kinase II. The incubation of cells for 4 h in 3% O_2_ resulted in a 50-fold increase in XOR phosphorylation, which increased the activity of XOR more than 2-fold. The increase in the XOR enzyme activity was not caused by the change in the amount of protein, but only by a posttranslational modification of the protein [89]. Furthermore, Poss et al. also noticed a 2-fold increase in XOR activity without changing the protein expression in bovine aortic endothelial cells incubated under hypoxic conditions (3% O_2_) [90]. However, the quantification of the activity and level of XOR expression in the lungs of rats exposed to a hypobaric atmosphere (0.5 atm) for 24 h showed that, in the lungs of those rats, the activity of XOR increased 2-fold, which was correlated with a 2-fold increase in the XOR mRNA and protein expressions determined by the PCR method and Western blot, respectively [90].

The proven overexpression (in vivo studies) or increased activation of xanthine oxidase (in vivo and in vitro studies) under hypoxic conditions suggests that nitro compounds as prodrugs might be metabolized by this enzyme, in addition to nitroreductase. In the 1990s, research had already been conducted concerning the reduction of nitroimidazole catalyzed by XO under anaerobic conditions [137]. The influence of XO as one of the possible or main enzymes activating compounds in hypoxia has been considered in many studies in recent years [138,139,140,141,142,143,144,145].

### 4.5. Cytochrome b_5_ Reductase (CYB5R)

Cytochrome b_5_ reductase (CYB5R) is a flavoprotein oxidoreductase enzyme that catalyzes the one-electron reduction of ferricytochrome b_5_ from (Fe^3+^) to ferrocytochrome b_5_ (Fe^2+^) using NADH as a coenzyme [146]. This enzyme exists in two isoforms that differ in location and function. The amphipathic microsomal isoform, together with cytochrome b_5_, is involved in the metabolism of xenobiotics, desaturation and elongation of fatty acids, cholesterol biosynthesis, and drug metabolism. The second, soluble form of CYP5R found in erythrocytes is responsible for the reduction of methemoglobin to hemoglobin [147]. Cytochrome b_5_ reductase, unlike the enzymes previously described, is present at relatively low levels, independently on tissue oxygenation. Apparently, for this reason, there is little interest in it, but several studies have confirmed that this enzyme plays an important role in activating some prodrugs [148]. It has been shown that, in addition to P450 reductase and quinone oxidoreductase, CYB5R is involved in the activation of the mitomycin C prodrug by reducing its quinone moiety. Its overexpression in cells increases susceptibility to the prodrug in relation to the parental line, both under aerobic and hypoxic conditions [149,150]. Other studies have revealed that CYP5R together with OX participates in the one-electron activation of the nitro-reduction group of KS119. Their overexpression in cells activates the cytotoxic effect of the compound analogously to type-II NTR, only under hypoxic conditions [140].

### 4.6. Critical Implications

The data presented in this section should be approached with some caution. Due to the very few reports on the examination of the level or activity of the enzymes listed in Table 2, driven by a direct comparison of cells cultivated under hypoxia to normoxia, they do not constitute statistically significant data concerning oxidoreductases involved in the activation of HAPs. These are selective data for only a few cell lines and, undoubtedly, in order to be able to draw meaningful conclusions, further research is needed. Recent extensive studies showed that 1-electron reduction is critically important in reducing HAPs and, among the enzymes involved in this process, cytochrome p450 reductase, followed by 5-methyltetrahydrofolate-homocysteine methyltransferase as well as NADPH-dependent diflavin oxidoreductase 1 (NDOR1), nitric oxide synthase 2 (NOS2), or cytochrome b_5_ reductase (CYB5R), were identified [76,121,151]. It should be noted that the type of oxidoreductase involved in the reduction of different HAPs may vary. Therefore, the profiling of the reductases relevant to each HAP, along with the assessment of their expression level or activity in tumors, would help in the stratification of patients. Although 2-electron oxidoreductases, such as NQO1 and aldo-keto reductase family 1 member C3 (AKR1C3), represent the “off-target” activation of HAPs due to their presence in normal human tissues, they can still serve for bioimaging since it relies on non-toxic metabolites and these enzymes are overexpressed in some tumors.

## 5. Visualization of the Level of Hypoxia

One of the main reasons for the failure of HAPs in clinical trials is the insufficient knowledge of the tumor oxygenation level in treated patients. Due to the high heterogeneity of tumors, the level of hypoxia can significantly differ in patients, even with the same type of tumor. Therefore, there is an urgent need to assess the level of hypoxia. The quantification of hypoxia enables the assessment of disease severity, the impact of therapy, and the patient’s prognosis. To date, numerous methods have been developed to selectively detect hypoxia in a clinical setting, such as Positron Emission Tomography (PET), Magnetic Resonance Imaging (MRI), and Electron Paramagnetic Resonance Imaging (EPRI) [152,153]. Nowadays, the most common non-invasive method to quantify tumor hypoxia is PET, which uses radiotracer probes that are selectively trapped in areas of hypoxic tissue [154]. Usually, the probes are ^18^F-containing 2-nitroimidazole (e.g., FMISO ((2-nitro-1-H-imidazol-1-yl)-3-fluoro-2-propan-2-ol), FAZA (1-(5-fluoro-5-deoxy-a-D-arabinofuranosyl)-2-nitroimi- dazole), or EF5 (2-(2-nitro-1H-imidazol-1-yl)-N-(2,2,3,3,3-pentafluor-opropyl)acetamide)), which can strongly covalently bind to cellular macromolecules in a hypoxic environment [152]. Additionally, ^60/64^Cu thiosemicarbazone derivatives (^60/64^Cu ATSM) and ^18^F fluorodeoxyglucose (18F-FDG) have been recently investigated as PET tracers [155].

One of the most promising hypoxia markers is EF5, which can be used both for hypoxia measurements by immunohistochemistry and flow cytometry, as well as hypoxia-specific PET imaging agents. Importantly, the activation of EF5 is strongly correlated with tissue oxygen concentration and oxidoreductase levels, making this compound a dual biomarker of hypoxia and oxidoreductases [14,17,18]. Several trials have confirmed that the covalent binding of the hypoxia-activated EF5 probe under hypoxic conditions correlates well with the activation of HAPs (e.g., SN30000 or Tirapazamine), which enables the use of the probe to predict the success of HAP therapy [56,121,156].

Another leading method for visualizing tissue hypoxia in human tumors is Blood Oxygen-Dependent Magnetic Resonance Imaging (BOLD MRI), which relies on the paramagnetic properties of deoxyhemoglobin to enable the assessment of blood oxygenation. The main limitation of this method is the imaging of the changes in blood oxygenation, which does not necessarily reflect the tissue oxygen level. The use of ^19^F- or ^31^P-labeled contrast agents enables the quantitative mapping of hypoxic tissue and is a promising alternative to ^1^H MRI [153]. Another very sensitive and direct method of quantifying tissue oxygenation is based on EPR spectroscopy. The measurement of oxygen concentration using the EPR oximetry method involves the use of an external paramagnetic probe that interacts with an unpaired oxygen electron, causing a change in the speed of spin–spin relaxation. The EPR technique is currently making significant progress, but there is still a need to design appropriate probes and instrumentation to be used in clinical trials [157].

### 5.1. Design Strategies for Fluorescent Turn off–on Probes for Hypoxia Imaging

Over the past decades, optical imaging has become one of the strongly developing methods of quantifying hypoxia. The considerable progress is due to the advantage of this technique, including the high sensitivity and low cost of probes, real-time monitoring, no use of ionizing radiation, nanometric resolution, and the possibility of direct intraoperative visualization [158]. Despite their many advantages, these methods still face many challenges. The use of optical imaging limits the depth of penetration; therefore, the development of methods is focused on near-infrared (NIR) fluorophores. NIR probes enable the deep penetration of photons into tissues and limit tissue damage. Additionally, the autofluorescence of endogenous tissue dyes does not disturb the signal of NIR probes [159].

**Figure 4:**
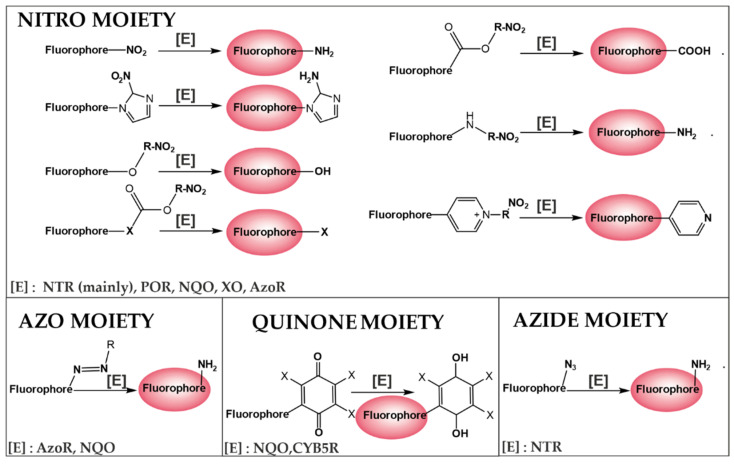
Overview of design strategies for fluorescent turn off–on probes for hypoxia imaging (based on ref [21]). [E]—oxidoreductase, NTR—Nitroreductase, POR—Cytochrome P450 reductase, NQO—Quinone oxidoreductase, XO—Xanthine oxidase, AzoR,—Azoreductase, CYB5R—Cytochrome b_5_ reductase, R—benzyl, imidazole, thiophene, or pyrrole moiety.

One of the approaches in the designing of hypoxia sensors is to use the reducing tumor microenvironment for irreversible reduction catalyzed by the overexpressed oxidoreductase. Small molecular probes for the detection of hypoxia, using the redox state of the cell, consist of a chromophore, a linker, and a hypoxia-sensitive moiety, i.e., the nitroaromatic, quinone, azobenzene, or azide derivatives (Figure 4) [160]. These moieties quench the fluorescence of the fluorophore emission and, upon reaching the target site that contains the appropriate oxidoreductase/s, the fluorescence is restored. The main mechanisms to turn off–on fluorescence include photoinduced electron transfer (PET), intramolecular charge transfer (ICT), excited-state intramolecular proton transfer (ESTPT), and Forster resonance energy transfer (FRET) [21,159]. The most widely developed group of small-molecule probes are compounds with a nitroaromatic group, which can be divided into two groups. The first consists of sensors based on the direct connection of the reaction group with fluorophores, which are reduced by NTR to the appropriate arylamine. The second group of NTR-sensitive compounds has a cleavable bond. In the presence of the enzyme, a cascade reaction occurs, leading to irreversible cleavage resulting in the release of free fluorophores. This type of probe mainly includes ether, benzylamino, ester, carbonate, or pyridinium linkage [21].

Apart from the nitroaromatic groups, azo derivatives can be another type of hypoxia-detecting moiety. Depending on the degree of hypoxia, the azo group can be reduced to aniline derivatives. Another group of fluorescent probes are compounds with a quinone moiety that can be converted into a hydroxyquinone catalyzed by overexpressed quinone oxidoreductase in the hypoxic microenvironment. Quinones are electron acceptors that effectively suppress fluorophore emissions [158]. Following the reduction, the resulting hydroquinones efficiently deliver electrons to the system, which restores fluorescence.

Detailed overviews showing the designed strategies for fluorescent probes, the underlying detection mechanism, and their potential applications have recently been reviewed in some excellent review papers [21,158,160].

### 5.2. Evaluation of NTR Expression under Hypoxic Conditions—Correlation between Fluoresent Imaging Probes and Biochemial Evaluation

Recently, much work has been conducted describing hundreds of probes, belonging to various group, activated by the reduction by oxidoreductases under hypoxic conditions. Unfortunately, most studies do not correlate the activation of the compounds with the expression or activity of these enzymes under the conditions studied [21,158,160]. In this chapter, we discussed the examples of fluorescent turn off–on probes whose emission signal is related to their reduction by nitroreductase under hypoxic conditions, and we compared their emission intensity to the expression of NTR.

**Figure 5:**
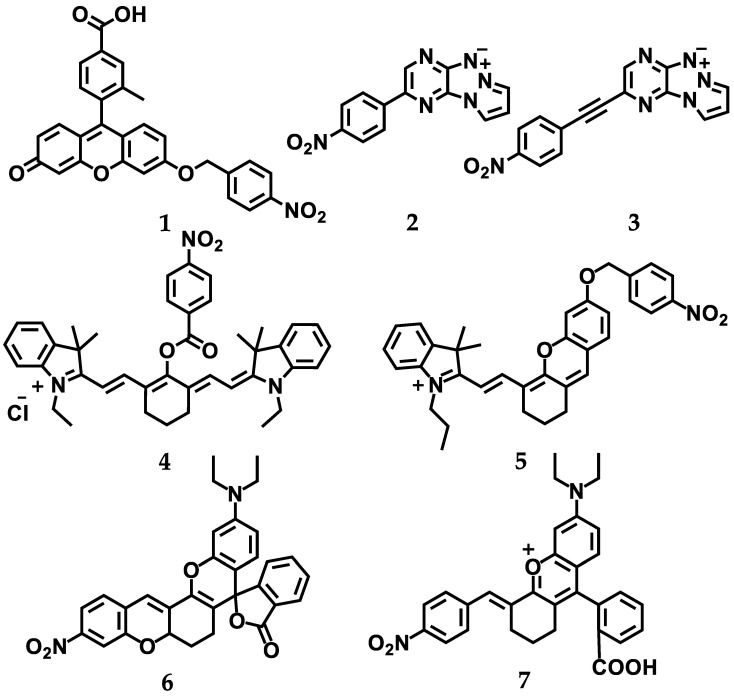
Structures of the selected hypoxia-activated fluorescence probes discussed in this review.

Luo et al. described a fluorescein probe coupled to a paranitrobenzyl group (**1**, see Figure 5) as a probe for the detection of hypoxia in tissue [80]. A free probe showing a weak fluorescent band, after incubation with NTR and NADH, was cleaved with the simultaneous release of a highly fluorescent derivative of fluorescein. In vivo studies performed for three cell lines, HepG-2, A549, and SKOV-3, showed a high correlation between the fluorescence intensity of the probe **1** and the degree of cellular hypoxia (15%, 8%, 5%, or 0.1% O_2_) determined by cytometry flow and confocal microscopy. The greater activity of the probe in hypoxia was not due to an increased level of NTR in cells, which was similar, but probably arose from the inhibition of the reoxidation of nitro anion free-radicals under hypoxic conditions. Moreover, it was shown that probe **1** could also be used to distinguish the growth stages of tumors that were correlated to the degree of hypoxia in in vivo models. Despite the constant level of NTR in tumors of various sizes, the fluorescence intensity differed more than 2-fold in a 14 mm tumor compared to a 7 mm tumor.

In 2021, our group, in cooperation with the group of Franck Suzenet, demonstrated that probes formed by the combination of a pyridazinetriazapentalene fluorophore with a hypoxia-sensitive nitrophenyl moiety (**2** and **3**, Figure 5) exhibited high sensitivity towards NTRs with a detection limit as low as 20–30 ng/mL [79]. Both compounds were effectively reduced by both type-I and -II nitroreductases to form highly fluorescent molecules. Tests conducted in the presence of an oxygen-sensitive type-II nitroreductase present in microsomes allowed for the completion of the reduction of the nitro derivative to its amine derivative under hypoxia, resulting in a high fluorescence signal, which was not observed under normoxic conditions. To the best of our knowledge, this work demonstrated, for the first time, the influence of a hydrophobic environment provided by the presence of human serum albumin (HSA) on the luminescent properties of compounds. HSA did not inactivate probe reduction, and the fluorescence intensity of the tested probes was strongly enhanced by the has in a concentration-dependent manner, which is a very desirable effect due to the cellular microenvironment. In addition, in vitro studies on the A2058 line showed a good correlation between the NTR level assessed by the ELISA test and these fluorescent turn off–on probes when determined under normoxia (21% O_2_), hypoxia (1% O_2_), and also chemically induced hypoxia (with desferrioxamine).

Li and co-workers developed five cyanine probes decorated with the nitro aromatic groups for the detection of NTRs, and, following protein docking and structural analysis, one molecule (**4**, Figure 5) was selected for further studies [82]. The fluorescence signal in the A549 cells incubated with probe **4** increased in proportion to the decreasing oxygen concentration (10%, 5%, 3%, and 1% O_2_); however, these results were not correlated with the level of NTR expressed in these cells. In vivo imaging in an A549 mouse tumor model using probe **4** showed a very rapid and accurate detection of the tumor, with confirmed hypoxia by PET imaging and expressed NTR by enzymatic detection. It was demonstrated that the probe could be used to distinguish the size of tumors varying in the degree of hypoxia and expression of NTRs. Additionally, it was possible to distinguish hypoxic tumors from inflammatory tissues. Importantly, due to the high penetration depth and low excitation power of the probes, the presented research may constitute a new promising strategy for the further development of novel probes for highly sensitive in vivo nitroreductase imaging studies.

Recently, Zhao Li et al. also conducted interesting research on a new near-infrared (NIR) fluorescent probe created by the conjugation of 4-nitrobenzene with the hemocyanin skeleton (**5**, Figure 5) for the imaging of nitroreductases in zebrafish in vivo [161]. The probe showed a high sensitivity to NTR in the solution; the detection limit was determined to be 14 ng/mL of nitroreductase. In addition, docking the probe to the protein revealed seven potential hydrogen bonds between the protein and the compound indicating a strong affinity. Moreover, in vivo studies on zebrafish correlated the level of endogenous nitroreductase in their organisms measured using the ELISA test with the fluorescence intensity of the tested probe. These results show that the probe has the potential to visualize the nitroreductase in in vivo models. In these studies, the hypoxia condition was not considered.

Near-infrared probes **6** and **7** (Figure 5) based on rhodamine fluorophore for NTR imaging in HeLa and HepG2 cells and in vivo were described by Zhang in 2022 [81]. It was shown that, in order to increase the sensitivity to NTR, it was necessary to limit intramolecular rotation, as in the case of probe **6**. The magnitude of the impact of the limited oxygen concentration in the atmosphere (10%, 5%, and 1% O_2_) was strongly correlated with both the NTR expression determined by the ELISA test and the increase in fluorescence. In addition, the probe was shown to be selectively accumulated in mitochondria and could be used to determine exogenous and endogenous nitroreductases in cells. The ability of the probe to be imaged in vivo was tested in the mouse HeLa tumor model. It was shown that the probe selectively accumulated after about 20 min only in tumor cells, and was removed within 6 h. The presented results indicate the potential of the probe for the quantitative imaging of hypoxic tumors in vivo.

These few examples show that there is no simple relationship between the fluorescence intensity of the probe and the presence of NTR. The observed signal is the superposition of two parameters, such as oxygen concentration and NTR activity, and their separation may be quite difficult. Therefore, such probes should be considered as combined sensors for imaging hypoxia and specific oxidoreductase states in cells or tissues. Another aspect that needs to be taken into account is the specificity of these probes to NTR. There is an urgent need for more intensive research to check the possibility of the activation of these sensors by other oxidoreductases to obtain a more complete image of their potential.

## 6. Conclusions

**Figure 6:**
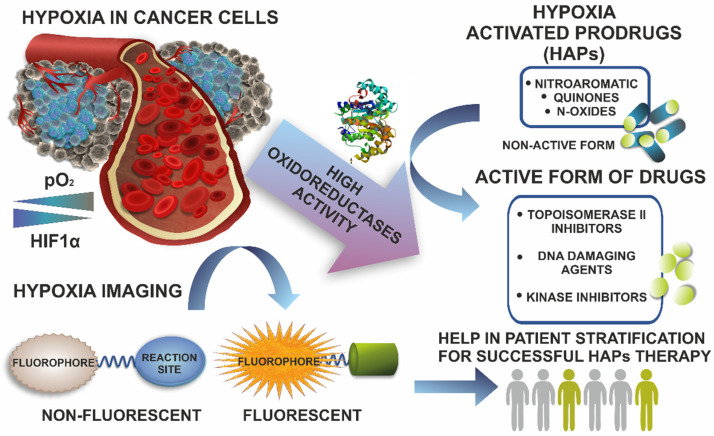
The overview of the role of oxidoreductases in hypoxia-activated prodrugs (HAPs) therapy and imaging of hypoxia.

Targeted therapy based on the features unique to specific cancer cells may develop thanks to our increasing knowledge about enzymes, proteins, or gene mutations that can drive cancer growth as well as our better understanding of the role of tumor microenvironment in its initiation, promotion, and progression (Figure 6). Hypoxia, as an important component of the tumor microenvironment, has attracted considerable attention from scientists to design therapies, which take advantage of this hallmark of rapidly growing solid tumors. Hypoxia-activated prodrugs (HAPs) that are selectively activated under hypoxic conditions, were developed and tested up to Phase III of the clinical trials. In spite of some encouraging antineoplastic efficacy in Phase II of the clinical trials, in Phase III studies, they showed little benefit and limited efficacy. As one of the possible reasons for the failure of HAPs in clinical studies, the lack of patient screening based on tumor hypoxia status was suggested. Therefore, the tools for the efficient and easy operating assessment of the oxygenation of cancer tissue are urgently needed. Furthermore, HAPs are activated by oxidoreductases, which have been presumed to be overexpressed under hypoxic conditions. However, as it was discussed in this review, our knowledge about the expression and activity of these enzymes in various cancer cell lines under hypoxic conditions is inadequate. The in vivo assessment of oxidoreductases is almost negligible.

The coexistence of two parameters, such as the presence of oxidoreductases and reduced oxygen concentration, are a prerequisite for successful HAPs therapies and patients expected to benefit from such therapies need to be pre-selected. Recently, the optical probes, which are based on turning on the fluorescence emission due to their irreversible reduction catalyzed by the overexpressed oxidoreductases, have been developed for the imaging of hypoxia. Such sensors are supposed to be very useful for assessing, in one step, both the activity of oxidoreductases and the degree of oxygenation. However, there is still insufficient information about the selectivity and sensitivity of such sensors. In spite of testing hundreds of sensors, only in a few examples, their response was correlated with the expression or activity of enzymes potentially responsible for turning on their emissions. In addition, to date, any routine procedure has been proposed to quantify the concentrations of oxidoreductases or degrees of hypoxia using such sensors in vitro, ex vivo, or in vivo. The available data rather focuses on the comparison of fluorescent emission of probes incubated with cell growth under different oxygen conditions. In vitro tests attributed to hypoxia were conducted under various conditions, such as the O_2_ concentration (varying from 0.1–3%) or incubation time, and various equipment was used to provide cells with appropriate conditions. Therefore, the comparison of the obtained data is not possible and the development of a more standard hypoxia model for this type of research would help in the evaluation of these sensors.

An alternative application of HAPs has been proposed by Li et al., who demonstrated that micrometastases (<1 mm in diameter) are highly hypoxic and therefore may be a target for this type of drug [1,162]. The group is currently working to prove this concept. This mode of action of HAPs would be particularly advantageous from the point of view of treating metastatic cancer at an early stage, for which no therapies are available.

## Figures and Tables

**Table 1:** Summary of clinical studies of HAPs (based on ref [38]).

Compound	Study Type	Target	Trial	Reference
Tirapazamine	Phase II	Squamous cell carcinoma of the head and neck	NCT00094081	[39]
			NCT00002774	[40]
	Phase III		NCT00174837	[41]
TH-302 (evofosfamide)	Phase III	Pancreatic cancer	NCT01746979	[42]
	Phase III	Soft tissue carcinoma	NCT01440088	[42]
	Phase III	Esophageal carcinoma	NCT02598687	[43]
Nimorazole (radiosensitizer)	Phase II	Squamous cell carcinoma of the head and neck	DAHANCA	[44,45]
	Phase III		NCT01950689	[46]
TH-4000 (tarloxotinib)	Phase II	Non-small cell lung cancer	NCT02454842	-
	Phase II	Squamous cell carcinoma of the head and neck	NCT02449681	[47]
PR-104	Phase II	Small cell lung cancer	NCT00544674	[48]
	Phase II	Non-small cell lung cancer	NCT00862134	
AQ4N	Phase I	Esophageal carcinoma	NCT00394628	[49]
	Phase II	Glioblastoma		
EO9 (apaziquone)	Phase III	Bladder cancer	NCT00598806	-
			NCT01475266	
			NCT02563561	
Porfiromycin	Phase III	Squamous cell carcinoma of the head and neck	NCT00002507	[50]

**Table 2:** Information on the evaluation of the expression or activity of specific oxidoreductases in vitro or in vivo under different oxygen concertation conditions using various biochemical methods. If not otherwise stated, cancer cell lines were used.

Specific Oxidoreductases	Detection Method	Cell Line	Enzyme Changes (↑ Increase Under Hypoxia)	Comment	Ref.
Nitroreductases (NTR)	NTR ELISA KIT	HepG-2	4 U/L (normoxia and hypoxia)	No significant change in NTR concentrations between cancer cells cultured at different oxygen concentrations	[80]
		A549	3 U/L (normoxia and hypoxia)		
		SKOV-3	2.5 U/L (normoxia and hypoxia)		
		HepG-2 (in vivo)	1.8 U/g (6 mm tumor diameter)		
			2.0 U/g (14 mm tumor diameter)		
	NTR ELISA KIT	A2058	180 pg/mL (normoxia)	Hypoxia led to the enhancement of NTR expression	[79]
			300 pg/mL (hypoxia)		
	NTR ELISA KIT	HeLa	~ 2× at 10% O_2_	Hypoxia led to clear enhancement of NTR expression	[81]
			~ 5× at 5% O_2_		
			~ 10× at 1% O_2_		
			compared to 20% O_2_		
	Western blot	A549 (in vivo)	No quantitative analysis (7 mm tumor diameter)	NTR expression only in tumor tissue	[82]
	Western blot detection of carbonic anhydrase 9 (CA9)	U87	**↑** ~ 2× at 2% O_2_	Indirectly assessing NTR activity by determination of CAIX	[83]
		U251	**↑** ~ 4× at 2% O_2_		
		GBM2	**↑** ~ 4× at 2% O_2_		
		GBM39	**↑** ~ 8× at 2% O_2_		
NAD(P)H quinone dehydrogenase 1 (NQO1)	Western blot	A549 H460	No quantitative analysis	Confirmed NQO1 expression, but not in normal cells (IMR90, HUVEC) No tests in hypoxia	[84]
		A549 (in vivo)		Confirmed NQO1 expression in tumor lysates, but not in other organs	
	Western blot	H460, HT-29, DU145, A549, FaDu, 9L, 9L/2B11, Colo-205, PC3, MCF-7, MB231, T47D, U251, BxPC-3, KM12, H522	No quantitative analysis	NQO1 levels were similar in cells grown under hypoxia (0.2% O_2_) and normoxia	[85]
	Northern blot	HT29	**↑** ~ 4× at 1 ppm O_2_	Hypoxia caused a marked increase in NQO1 level	[86]
Cytochrome p450 reductase (POR)	Western blot	UT-SCC-14	No quantitative analysis	Low expression of hypoxia and normoxia	[55]
		A549	No quantitative analysis	No change in expression of hypoxia vs. normoxia	
Xanthine oxidoreductase (XOR)	Western blot Northern blot (+XOR activity)	BEAS-2B	**↑** ~ 3× XOR activity	No changes in protein and mRNA expression under hypoxia	[87]
	PCR Western blot (+XOR activity determined by HPLC)	Rat lungs (in vivo)	**↑** ~ 2× (mRNA and XOR activity)		[88]
	Western blot (+XOR activity)	RPMEC (endothelial cells)	**↑** ~ 2.3× XOR activity	50-fold increase in phosphorylation, but without changing the OXR expression under hypoxia	[89]
	Western blot (+XOR activity)	BEAC bovine aortic endothelial cell	**↑** ~ 2× XOR activity	No changes in XOR mRNA expression under hypoxia	[90]

**↑** denotes increase in the expression or activity of specific oxidoreductases measured under hypoxic compared to normoxic conditions.
